# Effectiveness of a Social Media‐Based Life Review Intervention on Happiness in Advanced Cancer Patients and Their Family Caregivers: A Mixed‐Methods Study

**DOI:** 10.1002/pon.70374

**Published:** 2026-01-01

**Authors:** Wen‐Chi Yang, Chung‐Yi Li, Ching‐Liang Ho, Ping‐Ying Chang, Li‐Fen Wu, Hsueh‐Hsing Pan

**Affiliations:** ^1^ Graduate Institute of Medical Sciences College of Medicine National Defense Medical University Taipei Taiwan; ^2^ Department of Public Health College of Medicine National Cheng‐Kung University Tainan Taiwan; ^3^ Department of Public Health College of Public Health China Medical University Taichung Taiwan; ^4^ Department of Healthcare Administration College of Medical and Health Science Asia University Taichung Taiwan; ^5^ Taipei Tzu Chi Hospital Buddhist Tzu Chi Medical Foundation New Taipei Taiwan; ^6^ Division of Hematology‐Oncology, Department of Internal Medicine Tri‐Service General Hospital, National Defense Medical University Taipei Taiwan; ^7^ Department of Nursing Tri‐Service General Hospital Taipei Taiwan; ^8^ College of Nursing National Defense Medical University Taipei Taiwan

**Keywords:** cancer, caregiver, dyads, happiness, life review, media, mixed methods, nursing

## Abstract

**Objectives:**

This study evaluated the effectiveness of a social media‐based life review intervention on happiness in advanced cancer patient–caregiver dyads and explored participants' subjective experiences.

**Methods:**

A mixed‐methods design was employed in oncology wards of a medical center in northern Taiwan between September 2024 and January 2025. Sixty patient–caregiver dyads were recruited through convenience sampling and randomly assigned to an experimental (social media‐based life review) or control group (standard care). Final analysis included 28 dyads in the experimental group and 29 in the control group. Quantitative data were collected at baseline and post‐intervention using the Subjective Happiness Scale and analyzed with descriptive statistics, *t*‐tests, chi‐square tests, and generalized estimating equations (GEE). Qualitative data were collected through two consecutive semi‐structured interviews and were analyzed thematically to explore participants' experiences of happiness.

**Results:**

Patients in the experimental group maintained stable happiness, whereas those in the control group decline significantly (*p* = 0.023). Between‐group differences were non‐significant. Caregivers in the experimental group demonstrated a significant improvement in happiness (*p* = 0.025), with no change in control group. Patient happiness was positively associated with family support and self‐acceptance, whereas caregiver happiness was influenced by age, family support, and patient educational. Qualitative themes were *“Always being there is happiness”* and *“Rediscovering us through memories.”*

**Conclusion:**

Social media‐based life review intervention enhanced caregiver happiness and prevented decline in patient happiness, highlighting the potential of digitally mediated, family‐centered interventions in advanced cancer care.

**Trial registration:**

ClinicalTrials.gov identifier: NCT06559917.

## 1 Background

Cancer remains a major global health challenge and a leading cause of death worldwide. In 2022, nearly 20 million new cancer cases and approximately 10 million cancer‐related deaths were reported [1]. In Taiwan, cancer has been the leading cause of death since 1982, with 53,126 deaths reported in 2023, accounting for 25.8% of all deaths [2]. For patients with advanced cancer, the illness trajectory is often characterized by progressive functional decline, uncertainty, and profound existential distress [3]. Physical symptoms such as pain, fatigue, and nausea are often accompanied by psychosocial challenges, including anxiety, grief, and hopelessness [4].

Happiness is defined as a positive internal state arising from one's appraisal of life and emotions [5]. Evidence suggests that happiness is positively associated with self‐care ability [6] and that higher levels of happiness are linked to lower perceived symptom burden in patients with cancer [7]. Individuals with clearer goals tend to exhibit greater self‐acceptance, which is positively linked to subjective happiness [8]. Recently, happiness is recognized as a meaningful indicator of well‐being in advanced cancer care, influencing treatment engagement, symptom perception, and quality of life [9].

Life review refers to a structured process of recalling and narrating one's past, centered around personally meaningful themes and often facilitated by photographs, letters, scrapbooks, or mementos [10]. Common themes include family relationships, life transitions, education, and health. In addition to its educational value, life review serves therapeutic purposes by fostering self‐understanding and personal identity, while helping individuals cope with grief, and unresolved conflict, thereby reconstructing meaning and value in life [11]. Even brief interventions, such as a 1‐week life review, can enhance happiness and alleviate anxiety, depression, and psychological distress. For caregivers, sharing life review provides emotional support, reconciliation opportunities, and mutual understanding [12]. Thus, life review has the potential to improve patient–caregiver bonds and promote happiness for both parties during the end‐of‐life stage.

The application of social media in general life has become increasingly widespread, encompassing formats such as line groups, blogs, forums, and photo‐ and video‐sharing platforms. Their accessibility and flexibility have transformed information exchange and increasingly serve as adjuncts in healthcare [13]. For professionals, social media provides dissemination of knowledge and psychosocial support [14]. For patients with advanced cancer, social media engagement addresses barriers to face‐to‐face interventions, such as transportation difficulties, functional decline, and emotional reluctance [15]. Social media provides a familiar, flexible, and private environment in which patients can engage with supportive interventions at their own pace and within the comfort of daily routines [16]. Recent studies have examined the feasibility and effectiveness of social media‐based life review interventions. Chen et al. [17] implemented a 4‐week WeChat‐based life review program for 47 advanced cancer patient–caregiver dyads, reporting high acceptability and improvements in quality of life, family cohesion, and caregiver burden. Similarly, Zheng et al. [18] reported that a 4‐week WeChat‐based life review program for 150 patients with advanced gastrointestinal cancers significantly mitigated anxiety and depression, while enhancing hope and self‐transcendence. Despite these promising findings, the effect of social media‐based life review on happiness among advanced cancer patients and their primary caregivers has not been specially examined. Addressing this gap, the present study engaged dyads in a digital process of recalling and reconstructing personal and family experiences, aiming to foster emotional integration and mutual happiness.

Primary family caregivers play a pivotal role in end‐of‐life care, as patients with advanced cancer depend on them for daily assistance, symptom management, and emotional support. These contributions profoundly influence patients' quality of life and overall well‐being [19]. Caregivers' emotional states also influence patients' psychological adjustment, adherence to treatment, and overall coping, while reflecting caregivers' own adaptation to illness [20]. Given this interdependence, it is essential to examine patient–caregiver dyads as a unit of analysis. By enrolling both patients with advanced cancer and their primary family caregivers, this study aimed to capture the reciprocal influences within the caregiving relationship and evaluate the effectiveness of the intervention on the happiness of both groups. To capture both statistical outcomes and subjective perspectives, a mixed‐methods design was employed. Quantitative data from structured questionnaires measured change in happiness, while qualitative data from semi‐structured interviews provided insights into lived experiences. This approach ensured a comprehensive evaluation of both measurable effects and multi‐faceted personal meanings [21]. Therefore, the present study evaluated the effectiveness of a social media‐based life review intervention on happiness among advanced cancer patients and their family caregivers, and explored their subjective experiences following the intervention.

## 2 Methods

### 2.1 Design, Participants, and Setting

This study employed a mixed‐methods design and was prospectively registered with ClinicalTrials.gov (ID: NCT06559917). A dyadic approach was adopted, enrolling patients with advanced cancer and their primary family caregivers. Participants were recruited through convenience sampling from the oncology wards of a medical center in northern Taiwan between September 2024 and January 2025.

Eligible participants were dyads of patients with physician‐confirmed stage III or IV cancer and their primary family caregivers. Inclusion criteria for both patients and caregivers were: aged ≥ 20 years, full consciousness, ability to communicate in Chinese or Taiwanese, and no psychiatric history. Caregivers were defined as individuals with a biological or marital relationship who provided daily care and emotional support. Exclusion criteria for both groups included cognitive or language impairments, inability to follow study procedures, or involvement in medical disputes. Patients were excluded if sharing a hospital room with another enrolled participant. Caregivers were excluded if not directly involved in caregiving, had significant uncorrected sensory deficits, or lacked access or ability to use a smartphone and the LINE messaging application.

Sample size was calculated using G*Power 3.1, referencing a prior social media‐based life review study that reported an effect size of 1.28 [22]. Based on a two‐tailed test of independent means with α = 0.05 and 90% power, a minimum of 28 dyads was required. Allowing for 20% attrition [22], the target sample size was set at 34 dyads.

After providing informed consent, dyads were randomly assigned to the experimental or control group using a computer‐generated randomization list created by an independent statistician not involved in recruitment or data collection. A 1:1 allocation ratio was implemented. Group assignments were placed in sequentially numbered, sealed, opaque envelopes to ensure allocation concealment. Research personnel opened the next envelope only after the baseline assessment was completed, ensuring that allocation followed the predetermined random sequence without investigator influence.

### 2.2 Intervention

#### 2.2.1 Experimental Group

Participants in the experimental group received a social media‐based life review intervention. Each patient–caregiver dyad was given 1 month to collect a set of personal photographs that represented meaningful life events, such as family gatherings, travel experiences, major milestones, and everyday moments reflecting important roles or relationships. Dyads were asked to provide a sufficient number of photos to create a five‐minute life review video, which typically required approximately 20–50 photographs. These photos could be submitted in either digital or printed format.

Following photo collection, the researcher conducted a one‐on‐one session with each dyad to document the narratives, emotions, and contextual meanings associated with the selected photographs. These sessions lasted approximately 1–2 h. Using the photographs and accompanying narratives, the research team created a personalized five‐minute video incorporating soft piano background music. The same musical piece was used across all videos to ensure consistency in tone and emotional atmosphere throughout the intervention.

The completed video was then provided to the caregiver, who was instructed to view it with the patient and subsequently share it with other family members through the family's private LINE group. After the video was shared, dyads were free to rewatch or further share the video at their discretion; however, no researcher‐initiated follow‐up activities or additional interviews occurred during this period.

#### 2.2.2 Control Group

Participants in the control group received standard care provided during hospitalization, which included routine medical and nursing support such as vital‐sign monitoring, medication administration, symptom management, and assistance with activities of daily living. Primary caregivers continued their usual caregiving activities, including supporting meals, mobility, and treatment accompaniment, as well as offering emotional support as needed.

Ward nurses provided general health education, emotional support, and active listening consistent with routine clinical practice. The research team also offered brief supportive interactions, such as general encouragement and attentive listening, but did not deliver any structured psychosocial, educational, or life‐review components. This approach ensured that control group received usual care without exposure to intervention elements comparable to those in the experimental condition.

### 2.3 Instrument

Study instruments included basic characteristics of cancer patient and family caregiver, the Brief Symptom Rating Scale (BSRS‐5), the Subjective Happiness Scale (SHS), and a semi‐structured interview guide.

#### 2.3.1 Basic Characteristics

##### 2.3.1.1 Cancer Patient

Basic characteristics included age, gender, educational level (secondary or below vs. college or above), religious belief (no vs. yes), marital status (spouse vs. non‐spouse), cancer stage (III or IV), cancer duration (years), cancer treatment (chemotherapy only, chemotherapy with other treatments, other treatments, or no treatment), perceived family communication, support, and economic status (all rated good vs. poor). Self‐acceptance was defined as patients' acceptance of their illness and its implications, and life goals were defined as having an explicit purpose and personally meaningful aims for daily life and the future. These constructs were rated as either good or poor.

##### 2.3.1.2 Family Caregiver

Family caregiver data included age, gender, educational level (secondary or below vs. college or above), religious belief (no vs. yes), marital status (spouse vs. non‐spouse), perceived family communication, support, and economic status (all rated good or poor. Psychological stress was assessed using the Brief Symptom Rating Scale (BSRS‐5).

#### 2.3.2 The Brief Symptom Rating Scale (BSRS‐5)

The BSRS‐5 developed by Lee and colleagues at National Taiwan University, is a six‐item used to assess psychological distress experienced over the past week. Each item rated on a five‐point Likert scale (0–4). The first five items are summed to generate a total score ranging from 0 to 20, with higher scores indicating greater psychological distress. Standard cutoffs categorize distress as normal (0–5), mild (6–9), moderate (10–14), or severe (15–20). The sixth item assesses suicidal ideation separately to determine the need for referral for professional counseling. The BSRS‐5 demonstrates good reliability, with reported Cronbach's α values ranging from 0.77 to 0.90 and a 2‐week test‐retest reliability of 0.82 [23]. In this study, the BSRS‐5 showed a Cronbach's α of 0.86 at pretest across the full sample of 114 participants.

#### 2.3.3 The Subjective Happiness Scale (SHS)

The SHS, developed by Lyubomirsky and Lepper (1999), comprises four items rated on a 7‐point Likert scale to assess global subjective happiness. 1 indicates “not a very happy person,” and 7 indicates “a very happy person.” Scores range from 4 to 28, with higher scores reflecting greater happiness. Internal consistency has been reported between 0.79 and 0.94, with test–retest reliability 0.55–0.89 [24]. The scale was translated into Traditional Chinese and validated by Chien et al. (2020) in a sample of 543 university students, showing reliability, Cronbach's α 0.84, and test‐retest reliability of 0.89 [25]. In this study, the SHS was administered to a sample of 114 participants, comprising 57 patients and 57 family caregivers. Regarding the observed internal consistency, Cronbach's α in this sample for patients was 0.75 at pretest and 0.80 at posttest; for caregivers, it was 0.83 at pretest and 0.80 at posttest.

#### 2.3.4 Interview Guide

The semi‐structured interview guide was developed based on the research objectives included questions such as: “What does happiness mean to you?,” “How did this intervention influence your sense of happiness?,” and “What suggestions do you have for improvement?”.

### 2.4 Study Procedure

Data collection and study procedures were conducted between September 2024 and January 2025. The study followed a structured sequence from participant enrollment to intervention delivery and outcome assessment. Individuals who fulfilled the inclusion criteria were invited to participate, and written informed consent was obtained after explanation of the study objectives, procedures, and participant rights. Eligible dyads completed the baseline assessment prior to randomization.

Dyads were then allocated to either the experimental or control group using the computer‐generated randomization list. Dyads in the experimental group received a social media‐based life review intervention, whereas dyads in the control group received standard care.

Quantitative data were collected at two time points for both groups: at baseline (T1, pretest) immediately after enrollment and before randomization, and at posttest (T2), approximately 4 weeks later, after completion of the intervention period. In the experimental group, the posttest assessment was administered immediately after participants had viewed and shared the compiled social media–based life review content. All questionnaires were completed face‐to‐face using anonymous paper forms, and patients typically required 15–20 min to complete each assessment.

Qualitative data were obtained from dyads in the experimental group through semi‐structured interviews. The first interview, lasting approximately 1–2 h, was conducted during the intervention period when participants received and organized their selected photographs. The second interview, lasting about 60 min, was conducted following the posttest, after participants had completed viewing and sharing the life review content in the LINE group.

### 2.5 Statistical Analysis

Quantitative data were analyzed using IBM SPSS Statistics for Windows, Version 24.0 [26]. Descriptive statistics summarized characteristics, including frequencies and percentages for categorical variables and means with standard deviations for continuous variables. Baseline between‐group differences were assessed using independent *t*‐tests for continuous variables and Chi‐square tests for categorical variables. Within‐group SHS changes were tested using paired *t*‐tests, and between‐group differences using independent *t*‐tests. Generalized Estimating Equations (GEE) assessed the intervention effects over time and identified predictors of happiness.

Qualitative data were analyzed using the content analysis approach proposed by Graneheim and Lundman [27]. Transcripts were read repeatedly for immersion, then divided into meaning units. Subsequently, the transcripts were divided into meaning units, from which key words and phrases were identified. Thereafter, they were openly coded and grouped into subcategories based on their similarities, which were further abstracted into main categories. Throughout this process, the research team continuously compared the codes and categories with the original data to ensure accuracy and consistency. Finally, through an iterative process of comparison and abstraction, overarching themes and subthemes were developed to reflect the core experiences and meanings expressed by patients and caregivers.

### 2.6 Ethical Consideration

The study was approved by the institutional review board (IRB No. A202405117). All participants provided written informed consent after receiving full study information. Questionnaires were checked for completeness, securely stored, and anonymized. Personal data were destroyed after study completion to ensure confidentiality.

## 3 Results

### 3.1 Participant Characteristics

**FIGURE 1:**
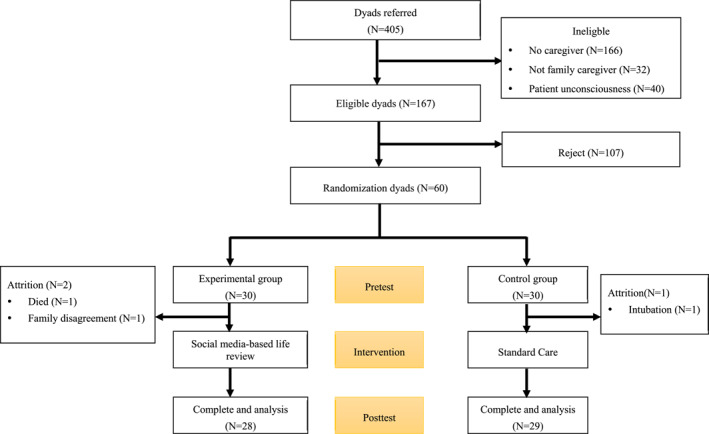
Consort flow diagram.

A total of 60 patient–family caregiver dyads were enrolled, with 30 dyads each assigned to the experimental and control groups. Three dyads were lost to follow‐up owing to patient death, caregiver withdrawal, or patient intubation. Finally, 28 dyads in the experimental group and 29 in the control group completed the study. The participant flow diagram is illustrated in Figure 1.

Basic characteristics did not differ significantly between groups regarding age, gender, educational level, religious belief, marital status, perceived family communication, support, economic status, psychological stress, cancer stage, duration, treatment, caregiver marital status, or life goals (all *p* > 0.05). The only significant was self‐acceptance (*p* = 0.009). Details are presented in Table 1.

**TABLE 1:** Basic characteristics of patients and their family caregivers.

Group	Patients			Family Caregivers		
	Experimental *N* = 28	Control *N* = 29		Experimental *N* = 28	Control *N* = 29	
	M ± SD/*N* (%)	M ± SD/*N* (%)	*p*	M ± SD/*N* (%)	M ± SD/*N* (%)	*p*
Age	63.1 ± 9.9	57.5 ± 15.1	0.060	55.7 ± 11.9	54.0 ± 13.2	0.575
Gender			0.735			0.429
Male	10 (35.7)	18 (62.1)		11 (39.3)	13 (44.8)	
Female	18 (64.3)	11 (37.9)		17 (60.7)	16 (55.2)	
Educational level			0.174			0.270
Secondary or below	19 (67.9)	17 (58.6)		11 (39.3)	15 (51.7)	
College or above	9 (32.1)	12 (41.4)		17 (60.7)	14 (48.3)	
Religious belief			0.331^#^			0.194^#^
No	4 (14.3)	8 (27.6)		1 (3.6)	5 (17.2)	
Yes	24 (85.7)	21 (72.4)		27 (96.4)	24 (82.8)	
Marital status			0.107			0.350
Non‐spouse	6 (21.4)	9 (31.0)		8 (28.6)	10 (34.5)	
Spouse	22 (78.6)	20 (69)		20 (71.4)	19 (65.5)	
Perceived family communication			0.458			0.286
Good	6 (21.4)	25 (86.2)		22 (78.6)	23 (79.3)	
Poor	22 (78.6)	4 (13.8)		6 (21.4)	6 (20.7)	
Perceived family support			0.906			0.857
Good	13 (46.4)	13 (44.8)		19 (67.9)	9 (31.0)	
Poor	15 (53.6)	16 (55.2)		9 (32.1)	20 (69.0)	
Perceived economic status			0.481			0.191
Good	7 (25.0)	5 (17.2)		6 (21.4)	8 (27.6)	
Poor	21 (75.0)	24 (82.8)		22 (78.6)	21 (72.4)	
Psychological distress	5.6 ± 4.7	3.6 ± 3.7	0.112	4.7 ± 3.9	3.2 ± 3.7	0.693
Cancer stage			0.350			
Stage III	8 (28.6)	10 (34.5)			
Stage IV	20 (71.4)	19 (65.5)				
Cancer duration (years)	3.2 ± 5.2	1.3 ± 1.2	0.057		
Cancer treatment			0.672		
Chemotherapy only	5 (17.9)	7 (24.1)				
Chemotherapy ± other treatments	18 (64.3)	17 (58.6)				
Other treatments	4 (14.3)	3 (10.3)				
No treatment	1 (3.6)	2 (6.9)				
Self‐acceptance			0.009		
Good	13 (46.4)	23 (79.3)				
Poor	15 (53.6)	6 (20.7)				
Life goals			0.243		
Good	14 (50.0)	19 (65.5)				
Poor	14 (50.0)	10 (34.5)				

Abbreviations: M = mean; SD = standard deviation; ^#^Fisher's Exact Test.

### 3.2 Effectiveness of the Intervention on Happiness

#### 3.2.1 Pretest‐Posttest Changes

For patients, no significant change in happiness was observed in the experimental group (21.6 ± 4.0 vs. 21.2 ± 4.8, *p* = 0.683), whereas the control group showed a significant decline (22.5 ± 4.0 vs. 20.5 ± 6.6, *p* = 0.023). Between‐group differences were non‐significant at pretest (*p* = 0.833) and posttest (*p* = 0.356).

For caregivers, happiness significantly increased in the experimental group (21.1 ± 5.4 vs. 22.7 ± 3.8, *p* = 0.025) but did not change in the control group (21.0 ± 4.2 vs. 20.5 ± 4.5, p = 0.306). Between‐group differences were non‐significant at both pretest (*p* = 0.670) and posttest (*p* = 0.184).

For dyads, no significant differences were observed between groups at either time point (*p* > 0.05). Results are summarized in Table 2.

**TABLE 2:** Effectiveness of the intervention on happiness among patients and family caregivers.

Item	Patients			Family caregivers			Patients vs. Family caregivers	
	Experimental	Control		Experimental	Control		Experimental	Control
	M ± SD	M ± SD	*p*	M ± SD	M ± SD	*p*	*p*	*p*
Happiness								
Pretest	21.6 ± 4.0	22.5 ± 4.0	0.833	21.1 ± 5.4	21.0 ± 4.2	0.670	0.504	0.568
Posttest	21.2 ± 4.8	20.5 ± 6.6	0.356	22.7 ± 3.8	20.5 ± 4.5	0.184	0.154	0.267
*p*	0.683	0.023		0.025	0.306			

Abbreviations: M = mean; SD = standard deviation.

#### 3.2.2 GEE Analysis

For patient happiness, multivariable GEE analysis showed no significant main effects of group (experimental vs. control, *β* = −0.19, 95% CI = ‐1.87 to 1.49, *p* = 0.824), time (Posttest vs. Pretest, *β* = −1.24, 95% CI = ‐2.54 to 0.06, *p* = 0.061), or group‐by‐time interaction (*β* = 0.88, 95% CI = ‐1.23 to 2.99, *p* = 0.411). However, good family support (*β* = 2.97, 95% CI = 1.09 to 4.85, *p* = 0.002), and self‐acceptance (*β* = 1.87, 95% CI = 0.12–3.62, *p* = 0.037) were positively associated with happiness. Patient life goals were not significantly related to happiness (*β* = 1.43, 95% CI = −0.28 to 3.14, *p* = 0.101). Detailed results are presented in Table 3.

**TABLE 3:** GEE model of patients' and family caregivers' happiness pre‐ and post‐social media‐based life review.

Variable	Adjusted			
	*β*	95% CI		*p*
Patient happiness				
Group				
Experimental versus Control	−0.19	−1.87	1.49	0.824
Time				
T2 versus T1	−1.24	−2.54	0.06	0.061
Group x Time				
Experimental xT2 versus Control xT1	0.88	−1.23	2.99	0.411
Patient family support				
Good versus Poor	2.97	1.09	4.85	0.002
Patient self‐acceptance				
Good versus Poor	1.87	0.12	3.62	0.037
Patient life goals				
Good versus Poor	1.43	−0.28	3.14	0.101
Family caregiver happiness				
Group				
Experimental versus Control	0.13	−2.05	2.31	0.910
Time				
T2 versus T1	−0.55	−1.57	0.47	0.288
Group x Time				
Experimental xT2 versus Control xT1	2.19	0.51	3.87	0.010
Family caregiver age	0.08	0.01	0.16	0.032
Family caregiver family support				
Good versus Poor	2.73	0.88	4.58	0.004
Patient education				
Secondary or below versus College or above	1.91	0.07	3.75	0.042
Patient cancer stage				
Stage IV versus Stage III	−1.06	−3.11	0.99	0.312
Patient disease duration (years)	0.12	−0.02	0.26	0.092

Abbreviations: GEE = generalized estimating equations; T1 = baseline assessment; T2 = post‐intervention quantitative data collection approximately 4 weeks after T1.

For caregiver happiness, GEE revealed a significant group‐by‐time interaction, with experimental group caregivers reporting higher posttest happiness (*β* = 2.19, 95% CI = 0.51 to 3.87, *p* = 0.010). Additional positively predictors included old caregiver age (*β* = 0.08, 95% CI = 0.01 to 0.16, *p* = 0.032), good family support (*β* = 2.73, 95% CI = 0.88 to 4.58, *p* = 0.004), and higher patient educational level (associate degree or above vs. high school or below, *β* = 1.91, 95% CI = 0.07 to 3.75, *p* = 0.042). The details are presented in Table 3.

Stratified analysis adjusting for caregiver age, family support, and patient characteristics (educational level, cancer stage, and disease duration) showed caregivers in the experimental group had a significant increase in happiness from pretest to posttest (*β* = 1.64, 95% CI = 0.31 to 2.98, *p* = 0.016). Control group caregivers showed a small, non‐significant decrease (*β* = −0.55, 95% CI = −1.57 to 0.47, *p* = 0.288). The findings are summarized in Table 4.

**TABLE 4:** Stratified analysis of the association between patient and caregiver demographics and caregiver happiness.

Basic characteristics	Adjusted			*p*
	*β*	95% CI		
Family caregiver group ‐experimental				
Time				
T2 versus T1	1.64	0.31	2.98	0.016
Family caregiver group ‐control				
Time				
T2 versus T1	−0.55	−1.57	0.47	0.288

*Note:* The model was adjusted for family caregivers' age, family support, as well as patients' educational level, and cancer stage.

Abbreviations: CI = confidence interval; T1 = baseline assessment; T2 = post‐intervention quantitative data collection approximately 4 weeks after T1.

### 3.3 Qualitative Findings: Happiness Experiences

Post‐intervention, 27 dyads participated in interviews guided by the semi‐structured tool. Two overarching themes were identified: first, *“Always being there is happiness”* and second, *“Finding us again through the memories.”* Corresponding subthemes for the first theme include, *“With you by my side, my heart feels at ease*” and “*In illness, the weight of love is truly felt.”* Corresponding subthemes for the second theme include, *“A gentle pull of memory keeps hearts from drifting apart,*” “*Looking back, the years we walked together still shine with light,”* and “*The shadow of the past reappears, and words flow from the heart.”* The details are presented in Table 5.

**TABLE 5:** Qualitative analysis of happiness experiences.

Theme	Subtheme	*Patient*	*Family caregiver*
1. Always being there is happiness	1. With you by my side, my heart feels at ease.	*“Our family goes to Pingxi every year to release sky lanterns. The next stop after Shifen Station is ‘Xingfu’ (happiness), so together it's ‘Shifen Xingfu’ (double happiness). We take pictures there every year.” (Patient01)* *“Our life is simple, peaceful, and happy. We don't cheat others, we don't have big worries—we just stay calm. I choose to live each day happily. It's all about mindset. Happiness is one day; joy is also one day—we choose joy every day.” (Patient10)* *“Happiness is being together. When everyone is united, we can move toward better goals, or at least share a common one.” (Patient11)* *“I'm currently living with my daughter‐in‐law, my grandson, and my daughter. I feel very happy living like this.” (Patient13)* *“To be honest, I've always been loved and spoiled—because I'm the youngest. My two older sisters treat me so well and take care of everything for me. I really have no worries. My second sister gives the most to our family. Usually, the unmarried ones give the most. She takes care of all of us before she takes care of herself. The happiness in our family comes from her.” (Patient17)* *“Freedom! Happiness is the freedom to make your own choices—what to eat, what to buy. As long as I have money, I buy a lot of model kits. No one controls me. That's my happiness—turning money into things I love!”(Patient18)* *“I feel happy just seeing people—especially children. When the whole family is together, that's true happiness.” (Patient26)*	*“The kids are all obedient and very filial. My daughter lives in Taipei—I don't even have to tell her when her dad is hospitalized. She comes by after work every day without being asked.” (Family02)* *“Happiness? I think even though I feel a lot of pressure because of the responsibilities, you could say we've gone through things others haven't. That, in a way, is a kind of happiness too. But it has also made me feel insecure about everything. That's the impact she (the patient) had on me. But what I've given her—she considers that happiness, because I've been with her from childhood until now.” (Family03)* *“We (my husband and I) always taught our children that the father is important—you think of Dad first, then Mom. ‘This piece is for Dad to eat’—even our grandchildren know this. So overall, I feel I'm quite happy. Our children are independent, and my husband and I have a good relationship.” (Family09)* *“I'm happiest when I'm with the kids. I love taking care of my grandchild—being with my grandchild is the happiest and most fulfilling thing for me.” (Family12)* *“Our happiness lies in the love between us as husband and wife. We've been married for over 30 years. Of course, couples argue sometimes, but I think overall, we're happy.” (Family15)* *“For me, happiness is the feeling of being needed. Like when I can cook for him, go with him to the doctor, and contribute to the family—that's what happiness means to me.” (Family18)* *“Mom always says she feels happy. She says she's lucky to have her daughter and son‐in‐law around her at this stage in life, always keeping her company. She feels very fortunate and happy.” (Family25)*
	2. In illness, the weight of love is truly felt	*“Now that I'm sick and getting older, I feel that I don't need to do anything every day. I just sit and draw, look at the flowers blooming in the courtyard, and I think that's already very good. The wind blows—it's already autumn and getting colder. Watching the seasons change and feeling it with my own body—that, to me, is happiness.” (Patient01)* *“Of course, I feel happier than before. After getting sick, people don't place as many expectations on you. You can basically do what you want, and more people care about you—people you didn't even interact with before, like former classmates or colleagues. Because of this situation, they now show extra concern and care.” (Patient20)*	*“I think happiness now needs to be lasting and enduring to truly count as happiness. If it's short‐lived, it just feels exhausting and powerless. Although this isn't the end stage of life yet, because we are still battling illness, it feels like happiness is limited—like it has an expiration date and cannot be extended.” (Family08)*
2. Finding us again through the memories	1. A gentle pull of memory keeps hearts from drifting apart	*“Yes! Every time I flip through photos and see how they celebrated my birthday every year, I can't help but smile as I look. Honestly, I think cancer is something we mostly scare ourselves with. There aren't many people as optimistic as I am!” (Patient21)* *“Even though it may seem like we're just looking for photos, when I see us taking pictures or eating together, I really feel a sense of happiness and cohesion. It reminds me that so many people care about us in everyday life. We often gather as a family, and every photo is a symbol of happiness—what gets captured are always the joyful moments.” (Patient26)*	*“I think it brought us closer. My mom rarely talked about things from my childhood—unless something specific triggered a memory, and even then, she'd only mention a few stories. But while looking for old photos, she started sharing more deeply. It let us relive the happiness from my childhood.” (Family24)*
	2. Looking back, the years we walked together still shine with light	*“I think looking through photos is a great way to reminisce. Whenever we go out, we have a group chat where we upload all the pictures. When we look at them, it brings back memories of what happened, and it gives everyone something to talk about.” (Patient11)*	*“They (referring to other family members) would recall things from the past, but I can barely remember them myself—some of those photos are just too old.” (Family03)*
	3. The shadow of the past reappears, and words flow from the heart	*“When I see photos of everyone gathered together, it reminds me that in daily life, we're all busy with everyday things—rice, oil, soy sauce, vinegar, and tea. From the moment we wake up, we're caught up in life. That's why taking a few days off to gather like this during holidays is such an important form of connection.” (Patient01)* *“While looking back at these photos and recalling those memories, I feel that life is better with them. Life feels more complete with their presence. I can care for them, and they care for me too. It's a kind of interaction, a mutual feeling of holding one another in our hearts.” (Patient23)*	

## 4 Discussion

### 4.1 Effectiveness of the Intervention on Happiness

This study indicated that advanced cancer patients who received social media‐based life review intervention maintained stable happiness, while control declined. Caregivers in the intervention group reported significant improvements, whereas those in the control group showed no change. These findings support prior evidence that psychosocial and reminiscence‐based interventions enhance well‐being in serious illness [28]. Unlike Thomas and Briggs [29], who compiled Facebook photos into printed “memory books,” our intervention created a video from personal photographs and shared it through a family LINE group. Both methods activate reminiscence via shared review and storytelling, but the digital‐video format offers unique advantages including replayability, remote co‐viewing, and rapid dissemination [30]. Although the number of video viewings was not formally tracked on the LINE platform, both patients and caregivers reported repeatedly watching the videos, which strengthened family connectedness and likely encouraged broader family interaction. This may explain why caregivers appeared to derive greater benefits from the intervention.

Intervention dose and timing are also crucial considerations. Zheng et al. [18] used a 1‐month WeChat‐based life review intervention with benefits lasting 6 months. Our single‐session, low‐dose intervention measured outcomes only immediately after completion. Nonetheless, it aligns with established short‐term life review protocols, typically two guided sessions with eight core questions and compiled materials for joint review, shown to promote meaning‐making, reduce anxiety and depression, and enhance self‐worth [31]. Caregivers may respond more strongly because they bear heavy emotional and practical burdens. Relationship‐focused interventions can provide immediate relief and affirmation; By contrast, patients' symptom load and psychosocial distress may limit short‐term gains [32].

### 4.2 Predictors of Happiness

Family support and self‐acceptance predicted patient happiness, while caregiver age, family support, and patient education predicted caregiver happiness. These findings echo Brajković et al. [8], who showed family support significantly promotes patient happiness. Although direct evidence linking self‐acceptance to happiness in cancer is limited, a concept analysis by Dwi et al. [33] concluded that higher self‐acceptance fosters psychological stability and adjustment, thereby enhancing happiness.

For caregivers, our findings are consistent with Zwar et al. [34], who found older caregivers more positive toward aging, aiding role adaptation. Song et al. [35] similarly reported that perceived family support strengthened emotional bonds and improved caregiver happiness. Education presented complex effects. Higher education is often linked to greater subjective happiness, yet Ge and Li [36] argued it may also increase anxiety from social comparison. Conversely, less educated patients may face reduced interpersonal stress but, as Zahedi et al. [37] noted, caregivers of less educated patients can experience lower preparedness. This suggests that while certain patient‐related anxieties might be mitigated, the practical demands or communication aspects could be perceived as more challenging. Therefore, when caregivers successfully navigate these potentially more demanding situations, their sense of accomplishment and role efficacy may be significantly strengthened, thereby enhancing their happiness.

### 4.3 Happiness Experiences

Qualitative findings revealed that dyads used shared photos and stories to revisit meaningful life events, recall childhood memories, and feel supported by family. This process promoted reflection and connection, with photos serving as symbols of happiness. Two themes captured these experiences: *“Finding us again through the memories”* and *“Always being there is happiness.”* Together, these themes highlight strengthened relational bonds, greater gratitude, and increased perceived happiness among both patients and caregivers.

Kwan, Chan, and Choi [38] identified a short‐term life‐review improved happiness but only modest affected anxiety and depression. Their booklet‐based intervention over two sessions differs from our single‐session, social media–based approach, yet both produced enhanced mood, gratitude, and renewed family perspective. Similarly, Miyawaki et al. [39] tested caregiver‐provided life review, with weekly sessions for 6 weeks using photos or objects. Results stabilized patient emotions, encouraged revisiting positive experiences, and improved relational quality. Our intervention achieved similar relational benefits despite being brief and digitally mediated.

These parallels reinforce life review as a means to strengthen connectedness and enhance happiness. Importantly, family involvement appears central. Hohashi and Yano [40] implemented a Family Life Review using the Plot of Family Story across two sessions. Participants reported stronger appreciation of family, openness in communication, and clarified shared memories. These findings align with ours: despite differences in delivery format and dose, life review consistently fosters relationship repair and deeper bonds, thereby enhancing perceived happiness.

### 4.4 Clinical Implications

This study highlights the potential of social media–based life review as a feasible, low‐cost, and family‐centered psychosocial intervention in advanced cancer care. For patients, such interventions may help preserve emotional stability despite disease progression. For caregivers, they provide meaningful opportunities to reflect, strengthen family bonds, and enhance well‐being. Given their simplicity and accessibility through widely used platforms, these interventions could be readily implemented in oncology and palliative care.

Clinically, nurses should systematically assess the psychosocial needs of both patients and caregivers, recognizing their interdependent experiences. Incorporating media‐based life review into routine care may foster emotional connection and mutual support within dyads. Holistic care must therefore extend beyond patients' biopsychosocial–spiritual needs to explicitly prioritize caregiver happiness, acknowledging their central role in end‐of‐life care. Broader adoption of digitally mediated reminiscence may strengthen dyadic coping, promote family cohesion, and support a more compassionate and comprehensive model of advanced cancer care.

## 5 Limitations and Recommendations

### 5.1 Limitations

This study has several limitations. First, it was conducted at a single medical center using convenience sampling within a randomized controlled trial, limiting generalizability to broader populations of advanced cancer patients and caregivers. Second, data were collected only at pre‐ and post‐intervention points, preventing evaluation of long‐term outcomes. Third, the intervention consisted of a single session due to time constraints, which may have reduced effectiveness compared with multi‐session approaches. Finally, only subjective outcome measures were employed, the absence of objective data may have weakened results robustness.

### 5.2 Recommendations

Future research should recruit from multiple institutions and regions to improve representativeness. Adequate staffing and double‐blind designs could further minimize bias. Longer follow‐up is recommended to examine sustained effects, and combining subjective assessments with objective outcomes may enhance validity and reliability.

## 6 Conclusion

This mixed‐methods study shows that a social media–based life review can help stabilize patient well‐being and significantly improve caregiver happiness. Qualitative themes, *“Always being there is happiness”* and *“Finding us again through the memories,”* highlight the emotional value of shared reflection and strengthened family bonds. Integrating digitally mediated life review into advanced cancer care appears feasible and family‐centered, offering a non‐pharmacological strategy to sustain patients' well‐being and enhance caregiver happiness.

## Funding

This work was supported by the Tri‐Service General Hospital, Taiwan (Grant TSGH‐E‐114298, TSGH‐A‐115016).

## Data Availability

The data that support the findings of this study are available on request from the corresponding author. The data are not publicly available due to privacy or ethical restrictions.
